# Application of the path analysis model to evaluate the role of distress, mental health literacy and burnout in predicting self-care behaviors among patients with type 2 diabetes

**DOI:** 10.1186/s13098-024-01375-z

**Published:** 2024-06-23

**Authors:** Alireza Jafari, Mahdi Moshki, Fatemehzahra Naddafi, Fatemeh Taghinezhad, Elham Charoghchian Khorasani, Negar Karimian, Zohre Farhadian, Hassan Alizadeh

**Affiliations:** 1 Department of Health Education and Health Promotion, School of Health, Social Development and Health Promotion Research Center, Gonabad University of Medical Sciences, Gonabad, Iran; 2 Department of Health Education and Health Promotion, School of Health, Social Development and Health Promotion Research Center, Gonabad University of Medical Sciences, Gonabad, Iran; 3 Student Research Committee, Gonabad University of Medical Sciences, Gonabad, Iran; 4 Social Determinants of Health Research Center, Mashhad University of Medical Sciences, Mashhad, Iran; 5 Department of Health Education and Health Promotion, School of Health, Mashhad University of Medical Sciences, Mashhad, Iran

**Keywords:** Diabetes distress, Health literacy, Self-care behaviors, Diabetes burnout

## Abstract

**Introduction:**

Mental complications of diabetes are one of the main obstacles to the implementation of self -care behaviors that have been less studied. Therefore, this study was conducted to survey the effective factors in predicting burnout and self-care behaviors among patients with type 2 diabetes.

**Methods:**

In this Path analysis, 1280 patients with type 2 diabetes were selected from Mashhad (Iran) in 2023 to 2024. Four scales, the mental health literacy (MHL) scale, diabetes burnout scale, diabetes distress scale, and self-care behavior scale were used for data gathering. AMOS software checked the direct and indirect paths between the variables.

**Results:**

In the path analysis, variables of MHL and diabetes distress predicted 25% variance of diabetes burnout (R^2^ = 0.25), and diabetes distress (total effect = 0.491) had the greatest impact on predicting diabetes burnout. Variables of MHL, diabetes distress, and diabetes burnout predicted 12% variance of Self-care behaviors (R^2^ = 0.12) and MHL (total effect = -0.256), age of onset of diabetes (total effect = 0.199), and diabetes burnout (total effect = − 0.167) had the greatest impact on prediction of self-care behaviors.

**Conclusion:**

MHL could reduce diabetes distress and burnout and eventually promote self-care behaviors among patients with type 2 diabetes. Therefore, screening and identifying psychological problems (such as distress and burnout) and designing interventions to increase MHL can ultimately increase the health of patients with diabetes.

## Introduction

Diabetes is a global danger to human health and is one of the major causes of mortality and morbidity worldwide [1, 2]. More than half a billion people in the world suffered from diabetes in 2021, and this number predicted to increase more than 1.3 billion by 2050 [3]. The results of the systematic review and meta-analysis study in 2024 also indicated the prevalence of 10.8% of type 2 diabetes (T2D) in Iran [2]. Diabetes is a lifelong and chronic disease with a defect in insulin function, insulin secretion, or both [1, 4]. Over time, the disease can also lead to numerous microvascular complications (such as retinopathy, nephropathy, neuropathy) and macrovascular complications (such as peripheral vessel disease, cerebrovascular accident, coronary artery disease) [5, 6].

Although diabetes control seems to be an unresolved problematic issue [3], self -care behaviors (SCB) could be an effective strategy for successful diabetes control and management [5]. Holistic SCB in diabetes include a healthy diet, blood sugar checks, regular medication, physical activity, correct problem-solving attitude, reduced risk factors, and maintaining healthy behavior [4, 7]. However, the results of a systematic review and meta-analysis study in 2023 showed that the level of SCB in people with T2D around the world is far from ideal [8]. In addition, the results of another systematic review and meta-analysis in 2021 in Iran showed a 48.86% rate of SCB in patients with diabetes, which is lower than the average [9].

The psychological complications of diabetes are one of the main obstacles to the implementation of SCB and have not been studied, contrary to the physical complications of diabetes [10–12]. Problems such as depression, anxiety, diabetes distress, and diabetes burnout are psychological complications of diabetes [11, 13–15]. In fact, managing and living with diabetes can be a complex and difficult task. Exposure to a large volume of SCB can make patients frustrated, discouraged, and angry, and ultimately weaken people’s motivation to perform SCB [12, 16] .

Initially, the burden of living with diabetes causes a sense of stress and guilt, which is called diabetes distress [17]. The prevalence of diabetes distress in India was 42% [18], 34.64% in China [19], 14% in Kuwait [20], and 48.6% in Iran [21]. The results of the systematic review and meta-analysis study also indicated the prevalence of 36% diabetes distress in the population of T2D patients worldwide [22]. According to numerous studies, diabetes distress is associated with reduced glycemic control and SCB [14, 20, 21].

Over time, permanent distress and emotional burden caused by diabetes management can cause exhaustion, frustration, detachment, and neglect of SCB; this is called diabetes burnout [15, 23]. A study conducted in 2020 indicated that the prevalence of moderate to severe diabetes burnout in type 1 diabetes was 50% in Iran and 22.57% in the United States of America [24]. Diabetes burnout can also cause negligent and even destructive SCBs in such a way that it leads to the labeling of non-adherent and incompatible titles for patients [15].

Although the psychological complications of diabetes could potentially diminish diabetes SCB, mental health literacy (MHL) can play a role as a protective factor [25]. Numerous studies have shown a positive and significant relationship between the levels of health literacy and SCB in patients with T2D [26, 27]. MHL is an effective factor for the early diagnosis and prevention of mental disorders [28–30]. MHL refers to the knowledge and skills of how to achieve mental health concepts, improve mental health, diagnose and treatment mental disorders, increase help efficiency, and reduce stigma related to mental disorders [31].The high MHL is associated with preventive activities, diagnosis of primary disorders, positive attitudes, and greater desire to seek mental health services [32]. Therefore, promoting MHL can help prevent and control the psychological complications of diabetes [25].

Overall, the psychological complications of diabetes, such as distress and burnout are the main obstacles to the implementation of SCB. However, promoting MHL can be a protective factor that enhances SCB [10–12, 25]. According to the abovementioned statistics, psychological complications of diabetes in the Iranian population are higher than other societies [21, 24]; however, the lack of studies in this regard, as well as the mere examination of direct relationships between these variables in the literature review, highlight the need to conduct more comprehensive studies. Therefore, this study was conducted using the path analysis method with two purposes in Mashhad, Iran:

1. Investigating the direct and indirect relationships among the variables of diabetes distress, diabetes burnout, MHL, and SCB.
2. Evaluating the role of distress, MHL and burnout in predicting SCB in T2D.

## Method

A path analysis study was designed and performed in 1280 T2D patients in Mashhad (Iran) in 2023 to 2024.

### Sample size

According to the previous study [33], the sample size was calculated as 1280 participants based on the following formula (test power = 80%, confidence level = 95%, prevalence of SCB = 31.9% had poor SCB, and accuracy/d = 0.04, 20% drop rate).

$$n=\frac{{({z}_{1-\frac{\alpha }{2}} +{z}_{1-\beta })}^{2} p(1-p) }{{\left(d\right)}^{2}}$$

### Sampling method

Participants were selected and entered the study using the method of proportional stratified sampling. Each comprehensive community health center in Mashhad city (*n* = 5) was considered as a stratum, and the population of T2D patients in each center was determined. In our community, patients with T2D are under the supervision of comprehensive community health centers and have an active health file in one of the centers. First, the list of all patients was extracted from the health file, and then people who had the inclusion criteria were determined. Subsequently, based on the sample size required from each center, people were selected by simple random sampling. When the selected people were referred to the centers for health services, the questionnaire was given to them and completed by self -report. In this study, data collection was conducted through paper surveys. If the person was not literate or it was difficult for the person to read the questions, the paper questionnaire was completed by the questioner and face-to-face interview. Inclusion criteria consisted of residence of more than one year in Mashhad city, had a health file in one of the Mashhad’s comprehensive community health centers, have T2D, diabetes duration more than 1 year, and being satisfied to participate in the study. In the analysis stage, people who did not respond to all questions and whose questionnaire had more missing data were deleted.

### Data collection scales

In this study, five questionnaires of demographic section, MHL scale, diabetes burnout scale, diabetes distress scale, and SCB scale were used for data gathering.

#### Demographic section

The variables of education level, marital status, occupation, economic status, sex, get information related to mental illness, sources of obtaining information related to mental illness, have another illness besides diabetes, refer to a health professional for mental-psychological, and etc. were surveyed.

#### MHL scale

This scale was designed and evaluated by O’Connor and Casey [34]. This scale was surveyed in the Iranian population and was confirmed, and Cronbach’s alpha coefficient was reported as 0.789. The Persian version of the MHL consists of 29 items with six subscales. The subscales were knowledge of risk factors and causes, knowledge of self-treatment, ability to recognize disorders, knowledge of the professional help available, knowledge of where to seek information, and attitudes that promote the recognition or appropriate help-seeking behavior. The minimum and maximum score of MHL is 29 to 145, and high scores show high MHL status [35].

#### Diabetes burnout scale

This questionnaire was designed in 2021 with the aim of determining the level of burnout in patients with diabetes. The status of burnout is a survey with 12 questions and three subscales: Loss of control, Detachment, and Exhaustion. Each item was measured with a 5-option Likert scale (“Completely agree” to “Completely disagree”). The minimum and maximum score of the diabetes burnout scale is 12 to 60, and high scores indicate high burnout status. Cronbach’s alpha coefficient in the original study was 0.80 [36] and in a study in Iranian patients with diabetes was 0.813 [37].

#### Diabetes distress scale (DDS)

This scale was created to determine the level of distress in patients with diabetes. The DDS includes 29 items and 2 sections of Sources of Distress and Core Level of Distress [38]. The part of the Core Level of Distress is measured with 8 items and the part of Sources of Distress is measured with 21 items. Also, in the Sources of Distress section, the status of seven subscales of Healthcare Access, Management Demands, Interpersonal Issues, Healthcare Provider, Long-term Health, Shame/Stigma, and Hypoglycemia were measured. All items were measured with a five-choice Likert scale, and the minimum and maximum score of DDS is 29 to 145, and high scores show high distress status [38]. Psychometric properties of DDS in Iranian patients with diabetes were evaluated, and Cronbach’s alpha coefficient for all items, part of Core Level of Distress, and part of Sources of Distress was 0.950, 0.914, and 0.920, respectively [39].

#### SCB (Self-care behavior) scale

It has 16 items and 4 subscales of glucose management, physical activity, dietary control, and health-care use. This tool was designed and evaluated by Schmitt et al. [40]. In the Iranian population, this tool has been approved by Nakhaeizadeh, and Cronbach’s alpha coefficient for this tool was 0.82 [41]. Each item was measured with a 5-option Likert scale (“applies to me very much” to “does not apply to me”). The minimum and maximum score is 16 to 66, and high scores show appropriate SCB [41].

### Statistical analysis

The relationship between the qualitative demographic characteristics and variables of MHL, diabetes distress, diabetes Burnout, and SCB were evaluated by independent samples t-test and One-way ANOVA in SPSS version 24. In addition, the correlation coefficients between variables were checked by Pearson correlation. The direct and indirect paths between the variables were assessed by the software of AMOS (version 24). For approval of the final model, the goodness of fit indexes of X2/df, RFI, CFI, GFI, IFI, RMSEA, NFI, TLI, and AGFI were checked [42–46].

## Results

The mean (SD) of diabetes duration, age of onset of diabetes, and age were 9.50 (7.30), 40.76 (12.17), and 50.37 (14.56) years, respectively. Most participants were male (*n* = 672, 52.5%) and housewife (*n* = 399, 31.5%). More patients with diabetes had an associate degree (*n* = 336, 26.6%), and only 12.8% (*n* = 160) had good economic status. Only 21.7% (*n* = 275) of patients were referred to a health professional for mental problems and only 43.2% (*n* = 115) declared that this visit was useful (Table 1).

**Table 1:** Frequency the characteristics of demographic variables

Variables			*n* = 1280	
			*n*	*%*
**Sex**	Male		672	52.5
	Female		607	47.5
**Marital status**	Married		1079	84.9
	Single		152	12
	Divorced		40	3.1
**Occupation**	Housewife		399	31.5
	Employed		244	19.3
	Retired		151	11.9
	Self-employed		386	30.5
	labor		68	5.4
	Unemployed		17	1.4
**Education level**	Illiterate		153	12.1
	Elementary		187	14.8
	Secondary		162	12.8
	High school		135	10.7
	Diploma		114	9
	Associate Degree		336	26.6
	Bachelor’s degree		165	13.1
	Master’s degree and more		9	0.7
**Economic status**	Good		160	12.8
	Medium		921	73.5
	Weak		172	13.7
**Get information related to mental illness**	Yes		802	63.2
	No		467	36.8
**Sources of information related to mental illness**	Physician/ Health care providers	Yes	97	7.6
		No	1183	92.4
	Psychologist/Psychiatrist	Yes	91	7.1
		No	1189	92.9
	Friends and acquaintances	Yes	72	5.6
		No	1208	94.4
	Book	Yes	155	12.1
		No	1125	87.9
	Internet	Yes	97	7.6
		No	1183	92.4
	Radio, television and satellite	Yes	339	26.5
		No	941	73.5
**Which type of diabetes complications are you currently experiencing?**	Eye complications	Yes	92	6.4
		No	1198	93.6
	Heart complications such as hypertension	Yes	364	28.4
		No	916	71.6
	Kidney complications	Yes	204	15.9
		No	1076	84.1
	Wound in one leg	Yes	42	3.3
		No	1238	96.7
	Wound in two legs	Yes	5	0.4
		No	1275	99.6
	Disconnect the organs	Yes	6	0.5
		No	1274	99.5
	Blood fat	Yes	687	53.7
		No	593	46.3
	All	Yes	2	0.2
		No	1278	99.8
**Do you currently have another illness other than diabetes?**	Yes		496	40.6
	No		715	58.5
	I do not know		12	1
**Refer to a health professional for mental-psychological**	Yes		275	21.7
	No		992	78.3
**Which specialist have you been referred for psychiatric problems?**	Psychologist/ Psychiatrist	Yes	133	10.4
		No	1147	89.6
	Physician	Yes	49	3.8
		No	1231	96.2
	Nurse	Yes	4	0.3
		No	1276	99.7
	Counselor	Yes	64	5
		No	1216	95
	Health care providers	Yes	56	4.4
		No	1224	95.6
**How helpful was it to visited a health professional for mental-psychological?**	Very useful		25	9.4
	Useful		115	43.2
	Low effect		112	42.1
	Very low effect		11	4.1
	Effectless		2	0.8
	I have no idea		1	0.4

Sex had significant relationship with diabetes distress, diabetes burnout, and SCB (*p* < 0.05). In addition, marital status, education level, economic status, and occupation had significant relationship with MHL, diabetes distress, diabetes burnout, and SCB (*p* < 0.05). People who were referred to specialists for mental disorders had low diabetes burnout and more SCB (*p* < 0.05) (Table 2).

**Table 2:** Relationship between demographic variables with MHL, diabetes distress, diabetes burnout, and self-care behaviors

Variables		*Mean (SD)*							
		**MHL**	P-value	**Diabetes distress**	P-value	**Diabetes burnout**	P-value	**Self-care behaviors**	P-value
Sex*	Men	76.50(6.06)	0.907	71.24(12.76)	< 0.001	32.70(4.86)	< 0.001	42.01(3.36)	0.009
	Women	76.55(7.03)		61.70(17.51)		29.89(6.64)		42.68(3.39)	
Marital status**	Married	76.35(5.93)	< 0.001	66.86(16.42)	0.036	30.62(5.65)	< 0.001	42.37(4.62)	< 0.001
	Single	80.32(7.16)		63.94(11.50)		34.36(5.65)		41.31(3.23)	
	Divorced	70.15(9.54)		70.27(16.68)		37.83(5.67)		44.30(2.76)	
Education level**	Illiterate	75.66(8.14)	< 0.001	59.69(18.35)	< 0.001	26.29(5.50)	< 0.001	43.92(7.16)	< 0.001
	High school or less	75.69(6.80)		66.88(17.17)		32.95(6.36)		43.18(4.22)	
	Academic	77.82(5.50)		68.76(12.39)		31.14(4.15)		40.67(2.63)	
Occupation**	Housewife	76.09(7.61)	< 0.001	59.89(17.61)	< 0.001	29.56(6.95)	< 0.001	43.84(5.55)	< 0.001
	Employed	77.88(3.24)		68.22(11.75)		30.50(4.27)		40.41(2.30)	
	Retired	75.86(5.24)		65.05(18.30)		30.34(7.50)		42.61(4.44)	
	Self-employed	75.87(7.27)		72.76(13.20)		33.60(4.27)		41.59(3.66)	
	labor	79.25(4.26)		70.72(10.12)		33.64(2.85)		43.85(3.90)	
	Unemployed	75.12(8.90)		66.52(8.75)		35.88(6.79)		42.68(3.49)	
Economic status**	Good	79.36 (7.74)	< 0.001	55.97(19.51)	< 0.001	28.24(8.04)	< 0.001	42.57(5.05)	0.001
	Medium	76.31(6.32)		69.34(13.67)		32.14(5.12)		42.54(4.45)	
	Weak	75.05(5.96)		63.02(18.43)		30.12(6.89)		41.18(3.76)	
Get information related to mental illness*	Yes	76.25(5.85)	0.058	67.93(14.13)	0.003	31.32(5.12)	0.481	42.18(4.51)	0.090
	No	77.02(7.55)		64.99(18.42)		31.58(7.05)		42.62(4.37)	
Refer to specialists*	Yes	75.64 (7.17)	0.014	66.22(14.76)	0.519	29.99(5.64)	< 0.001	43.80(5.89)	< 0.001
	No	76.82(6.31)		66.89(16.24)		31.74(5.89)		41.93(3.89)	
**Do you currently have another illness other than diabetes?**	Yes	75.23 (6.63)	< 0.001	66.21(16.08)	0.465	30.64(5.44)	0.101	43.59(5.04)	< 0.001
	No	77.90(5.59)		66.49(15.80)		31.34(5.90)		41.28(3.81)	
	I do not know	81.16(3.30)		71.93(18.92)		31.90(7.52)		44.74(3.54)	

* Independents sample T-test, ** One-way ANOVA

The mean (SD) of MHL, diabetes distress, diabetes burnout, and SCB were 76.53 (6.54), 66.70 (15.93), 31.37 (5.94), and 42.33 (4.45), respectively. Based on the Pearson correlation results in Table 3, MHL showed a negative and significant correlation with diabetes distress (*p* < 0.001, *r* = -0.187), diabetes burnout (*p* < 0.001, *r* = -0.113), and SCB (*p* < 0.001, *r* = -0.280). Diabetes distress showed a positive and significant correlation with diabetes burnout (*p* < 0.001, *r* = 0.483). In addition, diabetes burnout showed a negative and significant correlation with SCB (*p* = 0.001, *r* = -0.095) (Table 3).

**Table 3:** Pearson correlation between variables

Variables		MHL	Diabetes distress	Diabetes burnout	Self-care behaviors	Age	Age of onset of diabetes
**MHL**	Pearson Correlation	1	− 0.187^**^	− 0.113^**^	− 0.280^**^	− 0.220^**^	− 0.198^**^
	Sig. (2-tailed)		0.000	0.000	0.000	0.000	0.000
**Diabetes distress**	Pearson Correlation	− 0.187^**^	1	0.483^**^	0.014	− 0.184^**^	− 0.157^**^
	Sig. (2-tailed)	0.000		0.000	0.627	0.000	0.000
**Diabetes burnout**	Pearson Correlation	− 0.113^**^	0.483^**^	1	− 0.095^**^	− 0.033	0.022
	Sig. (2-tailed)	0.000	0.000		0.001	0.245	0.432
**Self-care behaviors**	Pearson Correlation	− 0.280^**^	0.014	− 0.095^**^	1	0.156^**^	0.197^**^
	Sig. (2-tailed)	0.000	0.627	0.001		0.000	0.000
**Age**	Pearson Correlation	− 0.220^**^	− 0.184^**^	− 0.033	0.156^**^	1	0.860^**^
	Sig. (2-tailed)	0.000	0.000	0.245	0.000		0.000
**Age of onset of diabetes**	Pearson Correlation	− 0.198^**^	− 0.157^**^	0.022	0.197^**^	0.860^**^	1
	Sig. (2-tailed)	0.000	0.000	0.432	0.000	0.000	
**Diabetes duration**	Pearson Correlation	− 0.110^**^	− 0.105^**^	− 0.116^**^	− 0.006	0.562^**^	0.073^**^
	Sig. (2-tailed)	0.000	0.000	0.000	0.820	0.000	0.009

**. Correlation is significant at the 0.01 level (2-tailed)

**Fig. 1:**
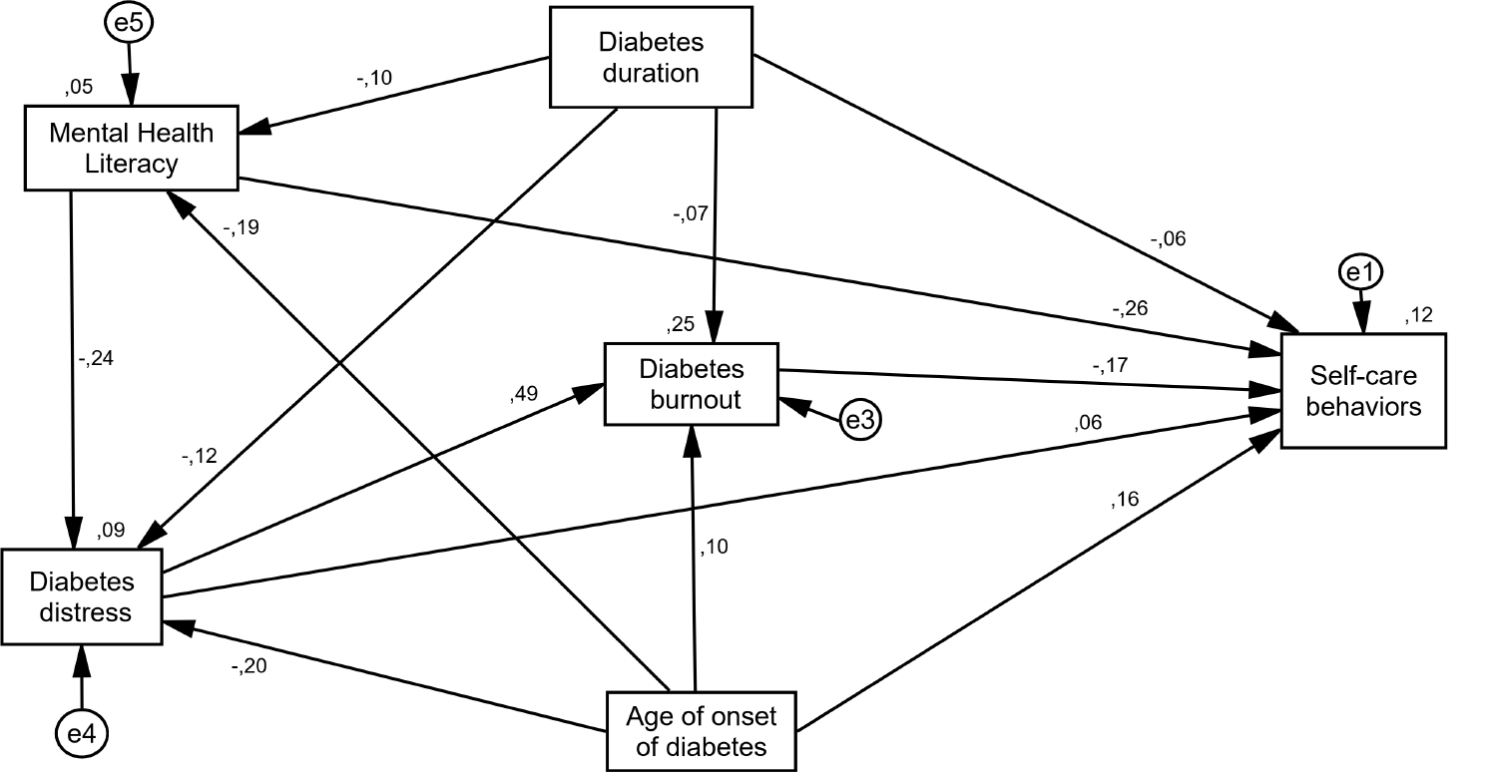
Direct and indirect paths between variables on predicting diabetes burnout (R^2^ = 25%) and self-care behavior (R^2^ = 12%)

In Table 4, goodness of fit indices (X2/df = 3.494, GFI = 0.998, CFI = 0.993, RMSEA = 0.044) approved the path model (Fig. 1). In Table 5, the indirect effects, direct effects, and total effects are mentioned, and MHL and diabetes distress predicted 25% variance of diabetes burnout (R^2^ = 0.25). In addition, MHL, diabetes duration, age of onset of diabetes, diabetes distress, and diabetes burnout predicted 12% variance of SCB (R^2^ = 0.12). In this study, standardized direct effects comprised 77% of the total causal effect and standardized indirect effects comprised 23% of the total causal effect. In the path model, the greatest impact on the prediction of diabetes burnout was related to diabetes distress (total effect = 0.491). In addition, the most impact in prediction of SCB was related to MHL (total effect = -0.256), age of onset of diabetes (total effect = 0.199), and diabetes burnout (total effect = − 0.167) (Table 5; Fig. 1).

**Table 4:** The model fit indicators of path model

Goodness of fit indices	Confirmatory factor analysis	Acceptable value
**X** ^**2**^	6.987	-
**df**	2	-
**X** ^**2**^ **/df**	3.494	< 5
**P-value**	0.030	> 0.05
**CFI**	0.993	> 0.9
**GFI**	0.998	> 0.9
**RMSEA**	0.044	< 0.08
**RFI**	0.927	> 0.9
**NFI**	0.990	> 0.9
**AGFI**	0.981	> 0.9
**IFI**	0.993	> 0.9
**TLI**	0.947	> 0.9

**Table 5:** Direct and indirect paths between variables

Determinants or Predictors	Standardized effects		
	Standardized direct effects	Standardized indirect effects	Standardized total effects
MHL ⟶ Diabetes distress	-0.239*	-	-0.239**
MHL ⟶ Diabetes burnout	-	-0.117**	-0.117**
MHL ⟶ Self-care behaviors	-0.260*	0.004	-0.256**
Diabetes distress ⟶ Diabetes burnout	0.491*	-	0.491**
Diabetes distress ⟶ Self-care behaviors	0.065**	-0.082**	-0.017
Diabetes burnout ⟶ Self-care behaviors	-0.167*	-	-0.167**
Age of onset of diabetes ⟶ Diabetes distress	-0.196*	0.046*	-0.150**
Age of onset of diabetes ⟶ Diabetes burnout	0.105*	-0.074**	0.031
Age of onset of diabetes ⟶ Self-care behaviors	0.164*	0.035*	0.199*
Age of onset of diabetes ⟶ MHL	-0.191*	-	-0.191**
Diabetes duration ⟶ MHL	-0.096*	-	-0.096**
Diabetes duration ⟶ Diabetes distress	-0.117*	0.023**	-0.094**
Diabetes duration ⟶ Diabetes burnout	-0.073**	-0.046**	-0.119**
Diabetes duration ⟶ Self-care behaviors	-0.060**	0.039**	-0.021
Total causal effect	0.574/0.746	0.172/0.746	0.746
Percantage of direct and indirects effects	77%	23%	100

MHL: Mental health literacy, *P < 0.001, **P < 0.05

## Discussion

This study was designed to investigate the potential psychological factors in predicting burnout and SCB among Iranian patients with T2D. Generally, the results showed that the variables of MHL, distress, diabetes duration, burnout, and age of onset of diabetes predicted 12% of the variance of SCB. Also, the variables of distress, age of onset of diabetes, MHL, and diabetes duration predicted 25% of the variance of diabetes burnout. As a result, diabetes onset at an older age, shorter diabetes duration, lower MHL and higher diabetes distress are associated with more diabetes burnout. In addition, the onset of diabetes at an older age, shorter duration of diabetes, and less diabetes burnout were associated with more SCB.

The results of the path analysis showed that diabetes burnout was one of the possible negative factors in SCB that could be significantly associated with a decrease in SCB in T2D. This result is in line with the results of previous studies [47–49]. According to a study by Kontoangelos et al., diabetes burnout occurs when the constant implementation of SCB causes physical and mental fatigue in patients and ultimately leads to neglect of the disease. As a result, one of the main consequences of diabetes burnout is physical fatigue and a sense of mental discharge to perform SCB [47]. In addition, in the qualitative study by Abdoli et al., reduced and abandoned SCB was one of the main results of diabetes burnout [48]. In a review study by Abdoli et al., it was found that diabetes burnout, in addition to poor therapy, can lead to serious problems such as depression and complications of diabetes in the long run and as a result, not only the sick person but also the family and caregivers are affected [49]. Therefore, given the potential negative effects of burnout on diabetes SCB, it is recommended that future studies examine further factors affecting diabetes burnout and develop interventions for decreasing burnout.

In our study, according to the results of path analysis, diabetes distress played the greatest role in predicting diabetes burnout. Diabetes distress is one of the most common mental problems among patients with T2D [50]. According to numerous studies, diabetes distress is a collection of negative emotions such as fear, anger, guilt, frustration, and if neglected, it will cause diabetes burnout [39, 50, 51]. A study on type 1 diabetes showed that Iranians had the highest diabetes distress and the highest diabetes burnout (distress: 57.1%, mean score of burnout: 3) compared with American patients (distress: 13.4%, mean score of burnout: 2.3) and Brazilian patients (distress: 30.8%, mean score of burnout: 2.6) [52]. In the study of Abd El Kader et al., in Egypt, it was found that SCB of diabetes can help to manage better and early diagnosis of distress; However, this relationship was not investigated in our study because of the limitation of path analysis in the investigation of two-way relationships [53]. In a study in Philippines by Totesora et al., it was found that there is no significant relationship between diabetes SCB and diabetes emotional distress [54]. The difference in the structure of the health system and cultural and social factors may be one of the possible reasons for the difference in the results. In addition, the questionnaire used to assess diabetes distress in our study was different from the study of Totesora [54]. In general, it seems necessary to carry out experimental studies to clarify these ambiguous relationships in the future. Overall, given the stable nature and high prevalence of distress as well as diabetes burnout in Iranian patients and the potential relationship between SCB, distress, and burnout, screening of patients with diabetes in terms of distress and burnout and referral to mental health professionals should be considered as a health priority.

MHL does not directly increase SCB, but by reducing diabetes distress could likely increase SCB. Means that higher MHL is associated with less distress and burnout and then ultimately more SCB. Therefore, in distress and burnout reduction, one of the factors that should be considered in programs is the issue of MHL so that we can ultimately promote SCB by increasing the level of literacy. In an interventional study by Vazifehkhorani in T2D, it was found that the implementation of cognitive behavioral therapy can enhance people’s MHL and thus improve their adaptation [55, 56]. In addition, poor MHL in diabetes patients was associated with decreased SCB and poor glycemic control [56]. The results of a systematic review and meta-analysis about psychological interventions for distress diabetes showed that different psychological interventions did not have a decisive effect on diabetes distress in the population with T2D compared to conventional care [57]. On the other hand, Cyranka et al., investigated the effect of a short-term psychological intervention on burnout and diabetes distress in a population of patients with type 1 diabetes, in Poland. The results indicated the significant effects of this intervention on reducing both diabetes burnout and distress [58]. These contradictions may be related to the difference in the type of interventions, duration of interventions, or socio-cultural factors and the structure of different societies. This issue highlights the need for more studies.

Various educational programs have been developed to promote diabetes SCB [59, 60]. Obviously, knowing the positive and negative factors affecting SCB is essential for diabetes SCB educators. Recognizing factors such as diabetes burnout and diabetes distress that have significant negative effects on the level of SCB can provide deeper insight to health care professionals to teaching patients. In this regard, developing more targeted educational programs and specific programs to reduce diabetes burnout and distress and improve MHL can be helpful. On the other hand, poor SCB is related to poor glycemic control [61], which is directly related to increased treatment costs, hospitalization, and lack of healthcare [62]. Therefore, by clarifying the effective factors in diabetes SCB, this study can be a basis for future longitudinal, experimental, and interventional studies, the development of targeted health education and health promotion programs, and future policy making of the health system.

The strengths of this study include a large sample size, use of standard tools, and determined of direct and indirect relationships between variables using path analysis. Among the limitations of this study, the following can be mentioned: conducting the study in only one city in Iran, this path analysis study only showed the relationships and it does not confirm causality. As a result, it is suggested that future studies with longitudinal and experimental designs to explain the causal relationships between these variables.

## Conclusion

This Path analysis study highlights the importance of screening and identifying psychological problems such as diabetes distress and burnout. Diabetes distress could potentially increase diabetes burnout, and eventually diabetes burnout will reduce SCB in patients with T2D. However, MHL can reduce diabetes distress and burnout as a Potential protective factor and eventually promote SCB in people with T2D.

## Data Availability

All data generated or analyzed during this study are included in this published article.

## Abbreviations

MHL: Mental Health Literacy
T2D: Type 2 diabetes
SCB: Self-care behaviors
AGFI: Adjusted goodness of fit index
x2/df: Chi-square ratio to degree of freedom
IFI: Incremental fit index
NFI: Normed fit index
RMSEA: Root mean square error of approximation
CFI: Comparative fit index
TLI: Tucker lewis index
GFI: Goodness of fit index
RFI: Relative fit index
